# Wholesome Flour

**Published:** 1876-10

**Authors:** 


In one of the last articles written by our colleague, the late Dr. Hall, he requested the lady readers of the Journal of Health to test the Cold Blast Whole-Wheat Flour as a bread-food, and to report the result. We have accumulated a good deal of correspondence on the subject, in answer to Dr. Hall's request, and are pleased to observe that the mass of testimony is strongly in favor of this superior flour.

The following is from a very intelligent and careful housekeeper, who has abandoned the use of all other flour for this more healthful and nourishing brand. It is evident that this improved flour is destined to become the well-nigh universal bread-food of such as seek the full worth of their money, and who recognize the fact that what is known as "Graham" flour is trashy, irritating, pernicious stuff, which has unquestionably done ten times more harm than good. We are in deep sorrow because of the death of a noble friend of ours who killed himself with crushed wheat, and "Graham," and other scouring trash. We wish we had the power to exterminate the pestilential stuff, whose only claim to value as food rests in the fact that it is mixed with nutritive substances, just as splinters of glass would be if sprinkled over our bread and butter. We entirely agree with Mrs. Chapman in her estimate of the value of the articles to which she alludes below:

Brooklyn, September 25, 1876.

"Dear Sir:—As any new article of food that promises an accession of health and strength is of importance to the community, it might be desirable for those who are testing it to present the results of their experience for the benefit of those who may not have heard of the article, and others who may be waiting for the public verdict upon it.

Before the introduction of the Health Food Company's Whole-Wheat Flour I regarded the cognomen, "staff of life," as applied to bread, as having more poetry than of fact in it; suggestive of what bread *ought* to be rather than of what it *is*. But made out of this *Whole-Wheat Flour*, I can at last realize that it is of a verity a staff of genuine support and sustenance; as from the increase of appetite and strength gained from the use of three-fourths of a barrel of this nutritious flour, I have reason to believe that it possesses more of life-sustaining and regulating properties than can be found in any other article of food in general use.

My family of seven all declare that it is the only kind of bread worth eating and worthy of the name; and I assure you we would all feel it a great privation to be compelled to return to the comparatively meager and unsatisfying article made from even the best of white flour.

As for the Pearled Oats made by this company, we think it would be quite impossible to find any better preparation of oatmeal at *any* price. Indeed, we think it fully equal at eight cents per pound to the best imported brands at twelve. The Pearled Wheat we find a most delicious breakfast dish; it also makes a pudding fully equal to the best tapioca.

In the beneficial effect following the use of the above articles, I realize, with deep but vain regret, the great value such foods would have been to me years ago in raising feeble and delicate children, establishing their health, and being thus spared many years of the most torturing and wearing anxiety on account of their extremely fragile condition. And I think that every mother in the land, who values a vigorous physical condition for her children, owes it to their future well-being, and can not commence too soon to feed them upon this Whole-Wheat Flour and these cereals to the exclusion of all other kinds less nourishing, in any form, whether as bread, cake, or pastry.

I have sent samples of my bread and cake from this flour to neighbors and friends, and the unanimous verdict is, "Delicious!" We regard the batter-and-crust fruit-puddings made from this flour as vastly superior in richness and fullness of quality to those made from any other. I have encouraged some of my friends to make a trial by sending them a sample of the flour; but the courage of some people is not equal to more than a first attempt in any other than white flour, though *I* have been singularly fortunate from the first in obtaining excellent results.

There are numerous recipes given for this bread in HALL'S JOURNAL OF HEALTH—a book no intelligent and health-seeking family should be without; but not having had time for experiments in them, I adhere to my own method, which is very simple and satisfactory. It is as follows: I sift from seven to eight pounds of the flour into a large bread-bowl, to which I add an even tablespoonful of salt and two heaping ones of ordinary sugar, which developes the delicacy and richness of the flour. I then add half a compressed yeast cake thoroughly dissolved in water, and about two quarts or a little over of water, blood warm. This will make five large loaves. I mix it with a large iron spoon until stiff enough to finish it with my hands—keeping them well floured and free from the dough—with light and dextrous kneeding until the whole is well-incorporated, yet rather soft—just stiff enough to leave my hands. In from three to four hours the dough will have risen sufficiently, in warm weather, to mold into loaves. As I think it best to bake only two at a time, and on the floors of the oven I have the latter in readiness, flour *well* a smaller bowl and my hands, and mold out two loaves with a quick movement, kneading as little as possible. When they have *commenced* rising in the pans, or in about ten minutes, I place them in the oven—which should be as hot as for white flour bread—when they finish rising as they bake; as, if they are too light before entering the oven, they will fall while baking and become coarse and uneven in the cell; while if allowed to rise only a little—say, one-third—in the pans, the result will be a fine, close, even cell, like pound cake, and a smooth, regular crust on the top. I find it very important to observe this point as a rule.

The baking requires from three-quarters of an hour to an hour, according to the heat of the oven, which should be uniform, but not scorching hot. The remaining dough I knead over a little harder to delay its action in rising too much before it can go in the oven; repeating this process as often as it is in danger of rising too much, and, consequently, souring. In making out the last loaves or loaf, I knead it barely enough to mold it, and consign it to the oven inside of three minutes.

I would remark that in my first attempts with this flour, not being an expert in handling the dough, which seemed to stick and melt, as it were, I found it necessary to use a little white flour to finish kneading the batch and for molding the loaves. As I became accustomed to handling it I was able to use less, and finally dispensed with it altogether. I can now manage it as easily as white flour. Beginners in the use of this flour will do well to have some white flour at hand to use as I have indicated. It is *necessary* to keep the hands or the molding-bowl or board *well* floured, kneading but little, and handling with a light and dextrous movement; otherwise, the dough, as I have said, appears to melt and sticks to the hand, until it seems a difficult matter to work in flour enough to finish the kneading.

I have been thus explicit in detailing all the minutiæ of my method for the benefit of those who may not have succeeded and would like to follow through all the steps, with the reasons and results of the process; and I am satisfied that those who once arrive at successful bread-making with this flour will never care to make use of any other.

Very truly yours, MRS. F. A. CHAPMAN,
931 Gates Ave., Brooklyn, N. Y.

---

The following comes to us from Mrs. Dr. Nelson L. North, of 91 Bedford Avenue, Brooklyn. We have been allowed to partake of the bread made by her from this recipe, and can testify to its appetizing qualities:

"91 BEDFORD AVENUE, BROOKLYN, N. Y.

"The way I make bread with the Cold-Blast Whole Wheat Flour does not differ materially from my usual mode with ordinary flour. To be sure this flour, in which is retained the gluten, as also many other of the nutritious properties so commonly destroyed or bolted out, is somewhat more 'sticky' than the old-way flour, yet I find no difficulty in overcoming that obstacle, through a little *vivacity* and *lightness* in the kneading.

"I first 'set a sponge' with some of this flour, using a pint of warm water to one half cake of compressed yeast, and flour enough to make a 'batter.' Set in a warm place, and in half an hour it will be raised, when I take five pounds of flour in my bread-bowl, open the middle, put in a tablespoonful of salt, a tablespoonful and a half of sugar, pour in my 'sponge,' and gradually add a sufficient quantity of warm water; I mix with the hands, kneading quickly and thoroughly, but not too stiff, using, from time to time, sufficient dry flour of the same kind to keep the hands free. I then cover and put in a warm place for two or two and a half hours. After it has risen, I make four loaves, and put in the pans with *very little molding*, and let them stand in pans in a warm place ten to fifteen minutes, when they are ready for the oven, which should be heated as usual for bread-baking. I bake about one hour. We find the bread delicious and very satisfying and nourishing.

Respectfully, MRS. N. L. NORTH."

---

Mrs. J. W. Rawlinson, of Port Chester, New York, makes a bread from WHITE WHEAT GLUTEN, which is characterized by Rev. J. Bastow of that place, as being delightful in flavor, quite porous and light, and a splendid building-up and brain-strengthening food. Here is the recipe which she sent us:

"For two medium-sized loaves make three pints of light sponge of half a yeast-cake and white flour or gluten, and water, and allow it to rise over night. Stir this sponge just before baking, together with four tablespoonfuls of brown sugar, two of butter, and one teaspoonful of soda, into about two pounds of gluten, or enough to make rather a soft dough, which should be slightly kneaded and molded into shape with the hands. Bake in a moderate oven, and the result will be splendid bread."

---

[*From Dr. Foote's "Health Monthly" for October*, 1876.]

THE HEALTH FOOD COMPANY.

There is something refreshing in the title, and something vastly more refreshing in the knowledge that the title fairly indicates the work accomplished by the organization bearing it. The theory of the company, condensed, is that diet should be intelligently varied, according to the bodily condition, the physical and mental wear and tear, and the state of the temperature. They learn from chemistry that nitrogen and carbon are demanded for the support of human beings. They observe that vast multitudes are weakened and debilitated by ignorantly employing one or the other of these elements in excess, and they make it their business to determine which constituent is lacking, and which is injuriously used. This fact known, the remedy for many ills of innutrition and imperfect digestion is found in restoring appropriate dietetic relations. They are not vegetarians alone, for they are aware that precisely the same food-elements are obtained from flesh, from grains, from fruits, and from roots, and they consider the variety in flavor an important factor in good digestion. Especially are they not "Grahamites," for they prove quite conclusively that "Graham" food — that is to say, food prepared from whole wheat or other grain, powdered or crushed—is a most dangerous aliment, sure to produce most devastating effects upon the stomach and bowels, if long continued, and therefore possessing in a marked degree the power to induce nearly all forms of disease, and greatly to shorten human life.

Every physician knows that sick people are very rarely quite right in the matter of digestion, and that until this function is well re-established the cure of other ills is tedious and difficult. We have not space in the MONTHLY to describe the various foods offered by this company for the relief and comfort of the stomach, or their new and improved methods of preparation of the cereals, whereby they are rendered more easy of digestion and more nutritious ; nor is it necessary that the space at our command should be devoted to this very worthy purpose, for the subject is already written up and published in a convenient form, which will be mailed gratuitously to all applicants who will take the trouble to address the Health Food Company at 137 Eighth Street, New York.

